# Evaluation of *in Vitro* and *in Vivo* Depigmenting Activity of Raspberry Ketone from *Rheum officinal*e

**DOI:** 10.3390/ijms12084819

**Published:** 2011-07-28

**Authors:** Chia-Hsiang Victor Lin, Hsiou-Yu Ding, Shiou-Yi Kuo, Ling-Wei Chin, Jiumn-Yih Wu, Te-Sheng Chang

**Affiliations:** 1 Department of Urology, E-Da Hospital, Kaohsiung 84001, Taiwan; E-Mail: victorlin0098@gmail.com; 2 The PhD Program of Biotechnology, Institute of Biotechnology and Chemical Engineering, I-Shou University, Kaohsiung 84001, Taiwan; E-Mail: wujy@isu.edu.tw; 3 Institute of Cosmetics Science, Chia Nan University of Pharmacy and Science, Tainan 71710, Taiwan; E-Mail: ab90377@hotmail.com; 4 Department of Biological Science and Technology, National University of Tainan, 33 sec. 2 Su-Lin St., Tainan 71702, Taiwan; E-Mail: seafly520@gmail.com

**Keywords:** B16 melanoma, tyrosinase, inhibition, zebrafish, melanogenesis, *Rheum officinale*, raspberry ketone

## Abstract

Melanogenesis inhibition by raspberry ketone (RK) from *Rheum officinale* was investigated both *in vitro* in cultivated murine B16 melanoma cells and *in vivo* in zebrafish and mice. In B16 cells, RK inhibited melanogenesis through a post-transcriptional regulation of tyrosinase gene expression, which resulted in down regulation of both cellular tyrosinase activity and the amount of tyrosinase protein, while the level of tyrosinase mRNA transcription was not affected. In zebrafish, RK also inhibited melanogenesis by reduction of tyrosinase activity. In mice, application of a 0.2% or 2% gel preparation of RK applied to mouse skin significantly increased the degree of skin whitening within one week of treatment. In contrast to the widely used flavoring properties of RK in perfumery and cosmetics, the skin-whitening potency of RK has been demonstrated in the present study. Based on our findings reported here, RK would appear to have high potential for use in the cosmetics industry.

## 1. Introduction

Raspberry ketone (4-(4-hydroxyphenyl) butan-2-one; RK), one of the major aromatic compounds of raspberry, is widely used in perfumery, in cosmetics, and as a flavoring agent in foodstuffs [1,2]. In 1965, the Federal Emergency Management Agency (FEMA) gave RK a “GRAS” (generally regarded as safe) status [3], and products containing RK have been marketed for weight loss in humans. RK has been confirmed to function as an anti-obesity treatment acting by stimulating the metabolism of white and brown adipose tissues and inhibiting absorption of dietary fat in the small intestine. RK exerts its anti-obesity effects via an increase of norepinephrine-induced lipolysis in white adipocytes and an enhancement of thermogenesis in brown adipose tissues [4]. A recent report showed that RK increased both lipolysis and fatty acid oxidation in white adipocytes [5]. In addition, RK also exhibits anti-androgenic activity [6] and anti-inflammation activity by blocking the nuclear transcription factor kappa-B activation pathway [7].

The skin color of animals and humans is determined mainly by the content of melanin pigment in the skin. Melanin is produced in dermal melanocytes by a process termed melanogenesis [8], which is initiated in special organelles within melanocytes, the melanosomes. Melanin synthesis begins with oxidation of l-tyrosine to l-DOPA (l-3,4-dihydroxyphenylalanine) and then to dopaquinone; both reactions are catalyzed by tyrosinase. The tyrosinase reactions are the rate-limiting step in melanin synthesis; the remainder of the reaction sequence proceeds spontaneously at physiological pH. Although melanin primarily serves a photoprotective function, the accumulation of abnormal amounts of melanin in different parts of the skin, which results in pigmented skin patches, can become an esthetic problem. Several studies have focused on inhibition of melanogenesis and the prevention of abnormal pigmentation for cosmetic benefits [9–11].

**Figure 1.**
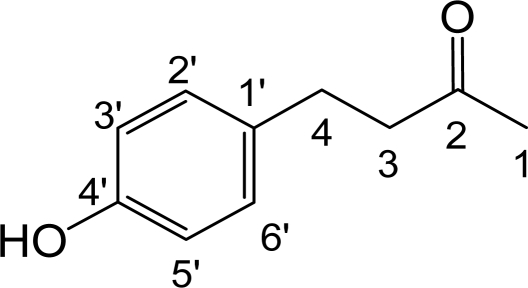
Chemical Structure of Raspberry Ketone (RK).

In our continuing search for melanogenesis inhibitors from natural compounds, we purified RK (Figure 1) from *Rheum officinale,* a traditional Chinese medicinal plant (family polygonaceae). In this study, the inhibitory effects of RK on melanogenesis were evaluated *in vitro* in cultured mouse B16 melanoma cells and *in vivo* in zebrafish and mouse model systems.

## 2. Results and Discussion

### 2.1. Purification, Identification, and Characterization of RK from R. officinale

In our preliminary study, we screened extracts from many Chinese herbs for new melanogenesis inhibitors and found that the ethanol extract of *R. officinal*e showed strong inhibitory activity against melanogenesis in B16 cells. Since melanogenesis inhibition by *R. officinal*e extracts has not been previously reported, we were interested in identifying the active compound. Bioassay-guided purification of the ethanol extract was carried out by methanol extraction, *n*-hexane, ethyl acetate, and water partitioning, and repeated silica gel column chromatography methods. Ultimately, one active compound was isolated. The chemical structure of the isolated compound was determined by mass spectra, ^1^H-NMR, and ^13^C-NMR analysis and its structure was resolved by comparing these data with those in the literature. The purified compound was identified as 4-(4-hydroxyphenyl) butan-2-one (raspberry ketone; RK); its chemical structure is shown in Figure 1.

RK is a major aromatic compound contained in red raspberries (*Rubus idaeus*), one of the oldest known fruits consumed by humans and used throughout the centuries for nutritional and medicinal purposes [12]. RK is found in many plants [13], but has not been previously isolated from *R. officinal*e.

### 2.2. Evaluation of In Vitro Depigmenting Activity of RK

**Figure 2.**
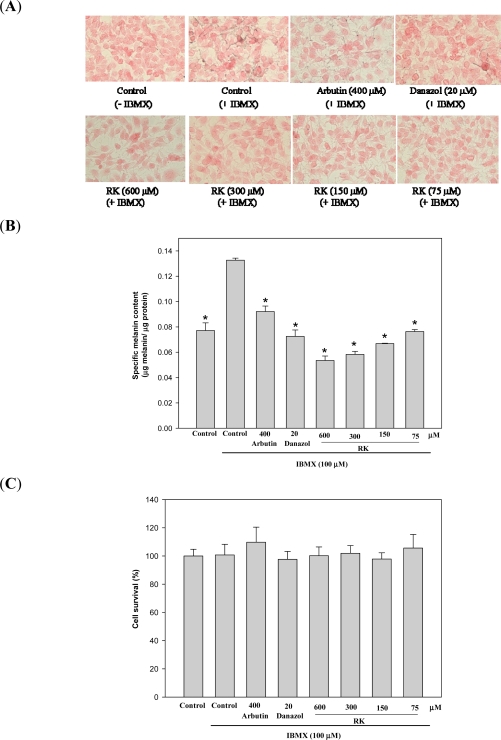
Effects of RK on Melanogenesis (**A**, **B**) and Cell Survival (**C**) in B16 Cells. The cells were cultivated for 1 d and then stimulated with 100 μM of IBMX for 2 d with various concentrations of RK. The melanin content of the cells was determined by Fontana-Masson staining (A) and spectrometry (B), as described in the Experimental Section, and cell survival was determined by the MTT method (C). Averaged data (n = 3) are presented with error bars indicating SD. A value of *p* < 0.001 (*), obtained with a Student’s *t*-test by comparing the data with those for the IBMX-stimulated control, was considered statistically significant.

In the present study, we used three systems to evaluate the depigmenting activity of RK, including *in vitro* cultured mouse B16 melanoma cell system and *in vivo* zebrafish and mouse models. The B16 cells were treated with RK and the melanin contents in the treated cells were directly monitored by Fontana-Masson staining of the cells. The results are shown in Figure 2A. In the present study, we used IBMX, which is an elevator of cellular cAMP level, to stimulate melanogenesis in B16 cells. Two melanogenesis inhibitors, arbutin and danazol, were used as positive standards in B16 cells [14]. The melanin content in the cells was increased after IBMX treatment and the increase in melanin content was reduced by both arbutin and danazol treatments. All RK treatments also significantly decreased the melanin content of the treated cells compared with that of the IBMX-stimulated cells. The melanin content of the treated cells was also determined by a photometric method, which detects the melanin content in cells via the absorption of the NaOH-dissolved melanin at 490 nm (Figure 2B). The resulting profile was similar to that obtained with Fontana-Masson staining and a dose-dependent melanogenesis inhibition by RK was clearly observed. Potential cytotoxic effects of RK on B16 cells were tested by treating the cells with varying concentrations of RK and measuring cell survival by the MTT method. As shown in Figure 2C, RK did not show any cytotoxicity although it strongly inhibited melanogenesis in mouse B16 melanoma cells.

**Figure 3.**
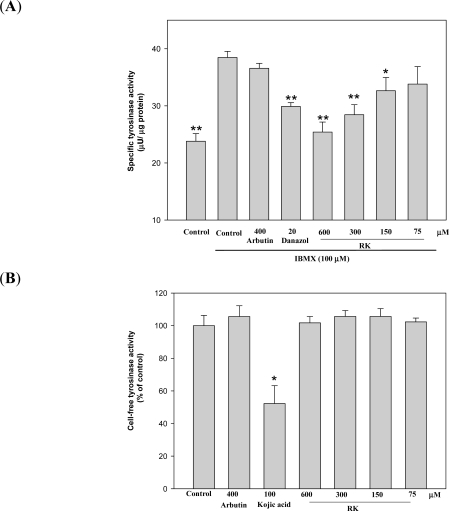
Effects of RK on Cellular (**A**) and Cell-free (**B**) Tyrosinase Activity in B16 Cells. Tyrosinase activity was determined by spectrometry, using l-DOPA as a substrate, as described in the Experimental Section. For the assay of cellular tyrosinase activity (A), B16 cells were cultivated for 1 d and then stimulated with 100 μM of IBMX for 2 d with various concentrations of RK. At the end of the drug treatment, the cells were harvested and assayed for the cellular tyrosinase activity by adding the enzyme substrate. For the assay of cell-free tyrosinase activity (B), untreated cells were lysed to obtain a crude tyrosinase extract. The cell-free tyrosinase activity was determined by directly mixing the crude tyrosinase, l-DOPA, and various concentrations of RK. Averaged data (n = 3) are presented with error bars indicating SD. A value of *p* < 0.05 (*) or *p* < 0.001 (**), obtained with a Student’s *t*-test by comparing the data with those for the IBMX-stimulated control (A) or control (B), was considered statistically significant.

Because tyrosinase plays a key role in melanogenesis, we then investigated the effects of RK on the activity of this enzyme using a photometric method (Figure 3A). RK treatment resulted in a dose-dependent reduction in the cellular tyrosinase activity. Therefore, melanogenesis inhibition by RK in B16 cells apparently occurred via a reduction in tyrosinase activity. The reduction in cellular tyrosinase activity by RK might be caused either by a direct inhibition of enzyme activity or by a reduction in the amount of tyrosinase protein in the cells.

**Figure 4.**
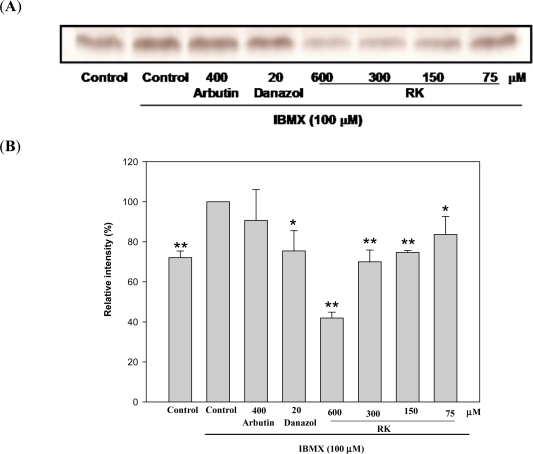

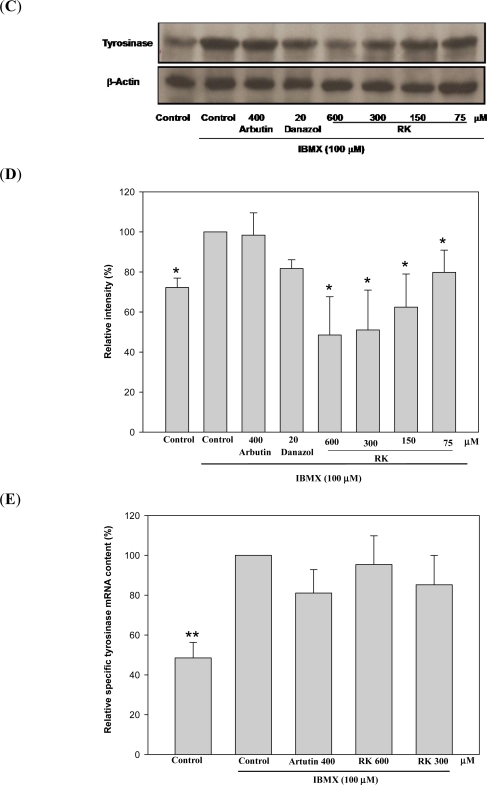
Evaluations of RK on the Effects on the Amounts of Tyrosinase Protein by Zymography (**A** and **B**) and Western blot (**C** and **D**) and Tyrosinase mRNA by qRT-PCR (**E**) in B16 Cells. Cells were inoculated in 24-well plates for 1 d and then stimulated by 100 μM IBMX with and without the test drug. They were harvested and the tyrosinase protein and mRNA were analyzed as described in the Experimental Section. The band intensities of tyrosinase from results of western blot and qRT-PCR were normalized by that of β-actin and GAPDH, respectively, and the normalized band intensity in the IBMX-stimulated control was recalculated to be 100. Averaged data (n = 4 for zymography and western blot; n = 7 for qRT-PCR) are presented with an error bar of SD. A value of *p* < 0.05 (*) or *p* < 0.001 (**) by Student’s *t*-test by comparing the data with that of the IBMX-stimulated control was considered statistically significant.

To confirm the first possibility, the effects of RK on enzyme activity were determined by assays of cell-free tyrosinase extracts (Figure 3B). RK did not directly affect the tyrosinase activity indicating that RK was not a tyrosinase inhibitor. The effects of RK on the amount of cellular tyrosinase protein were determined by both in-gel staining of tyrosinase activity (zymography) and western blotting analysis. The B16 cells were treated with RK and the total proteins in the cells were separated by gel electrophoresis. Tyrosinase activity in gels was stained by l-DOPA (Figure 4A and 4B) and tyrosinase protein in gels was recognized by an anti-tyrosinase antibody (Figure 4C and 4D). Both tests demonstrated that RK treatments significantly and dose-dependently reduced the amount of tyrosinase protein in the cells. RK therefore appears to inhibit melanogenesis of B16 cells by reducing the amounts of cellular tyrosinase, thereby decreasing the cellular tyrosinase activity.

One possible explanation for the reduction in protein amount in the cells would be inhibition of gene transcription. The effects of RK on tyrosinase gene transcription were examined by determining the amount of tyrosinase mRNA in the RK-treated cells by a qRT-PCR method (Figure 4E). Unexpectedly, RK had no effect on the amount of tyrosinase mRNA, even at as high a concentration as 600 μM. This suggested that RK inhibits melanogenesis through a post-transcriptional regulation of tyrosinase gene expression, which resulted in down regulation of both cellular tyrosinase activity and the amount of tyrosinase protein, while the level of tyrosinase mRNA transcription was not affected. Further molecular details of the mechanism of melanogenesis inhibition by RK in B16 cells need to be determined in the future.

### 2.3. Evaluation of In Vivo Depigmenting Activity of RK

**Figure 5.**
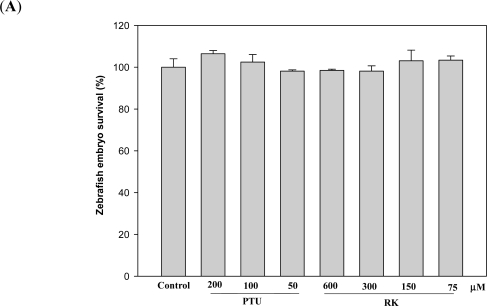

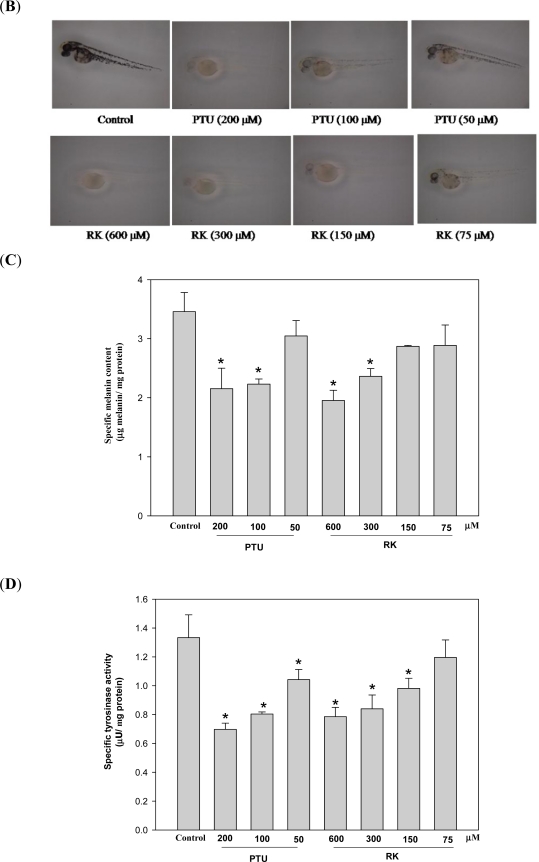
Evaluation of Depigmenting Activity of RK in Zebrafish. A total of 100 synchronized embryos were treated with drugs from 9 to 48 hpf (hours post fertilization), and normally developed embryos were collected to calculate embryo survival (**A**). Melanin pigment was photographed (**B**) and its quantity was determined by a photometric method (**C**), as described in the Experimental Section. For assays of tyrosinase activity (**D**), 250 μg of total protein from lysates of 100 zebrafish larval was incubated with 2.5 mM of l-DOPA, and the resulting dopachrome was quantified by a photometric method, as described in the Experimental Section. Averaged data (n = 3) are presented with an error bar of SD. A value of *p* < 0.05 (*) by Student’s *t*-test by comparing the data with those for control was considered statistically significant.

The zebrafish is a popular vertebrate model system in many research fields [15]. Recently, it was also established as a new *in vivo* model for evaluating the depigmenting activity of melanogenic regulatory compounds [16]. This animal model system has several advantages, including easy maintenance and handling of the animals and high efficiency of drug penetration through the skin. For these reasons, the zebrafish model was used as an *in vivo* system to evaluate the inhibition of melanogenesis by RK. The cytotoxicity of RK against zebrafish embryos was first determined (Figure 5A). RK showed no significant toxicity toward zebrafish embryos at the tested concentrations, up to 600 μM.

The melanin content of the RK-treated fish was recorded by photography (Figure 5B) or by a photometric method (Figure 5C). In both cases, the treatment of embryos with RK between 9 to 48 hpf (hours post fertilization) significantly and dose-dependently reduced skin melanin content in the developed larvae. At a concentration of 300 μM, RK caused the same degree of transparency as seen for 200 μM of 1-phenyl 2-thiourea (PTU), a standard depigmenting agent used in zebrafish experiments. In addition, RK also dose-dependently reduced the zebrafish tyrosinase activity (Figure 5D), indicating that RK reduced melanogenesis in zebrafish by suppression of cellular tyrosinase activity. No direct inhibition of the cell-free zebrafish tyrosinase activity was observed with RK treatment (data not shown). Thus, RK appeared to inhibit melanogenesis in zebrafish via a similar mechanism as that seen in the mouse B16 cells. However, due to the lack of a commercial anti-tyrosinase antibody for zebrafish, we could not confirm whether RK also reduced the amount of tyrosinase protein in zebrafish.

**Figure 6.**
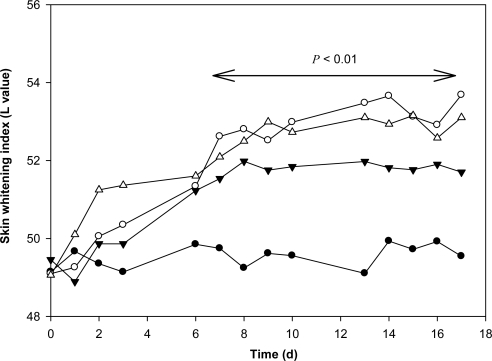
Time Course of Skin Whitening Index (L value) of Mice Treated with Vaseline only (control, •), 2% HQ (○), 0.2% RK (▾) or 2% RK (▵) in Vaseline. A total of thirty-two mice were equally divided into four groups and each group was smeared twice daily in 5 consecutive days a week for 3 weeks with 0.1 g Vaseline (control, 
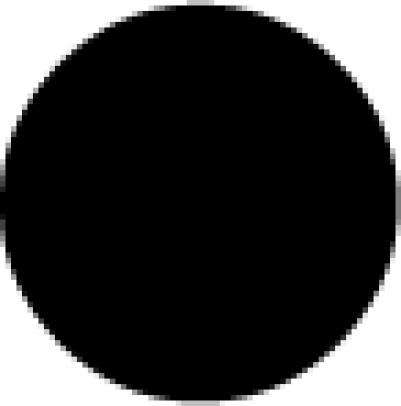
), 2% HQ in Vaseline ( 
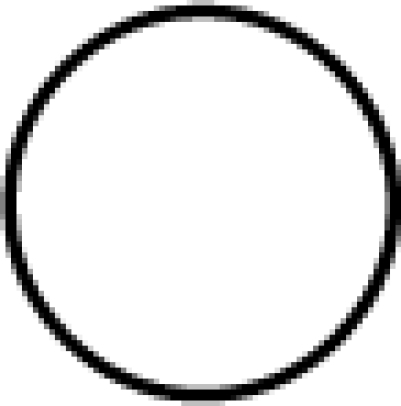
), 0.2% RK ( 
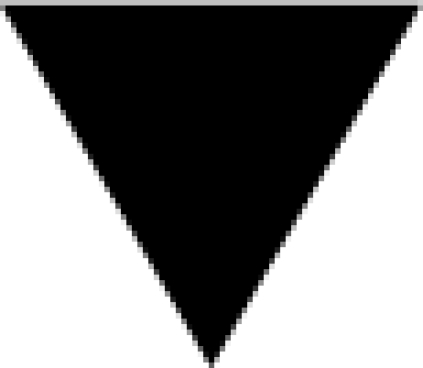
) or 2% RK in Vaseline ( 
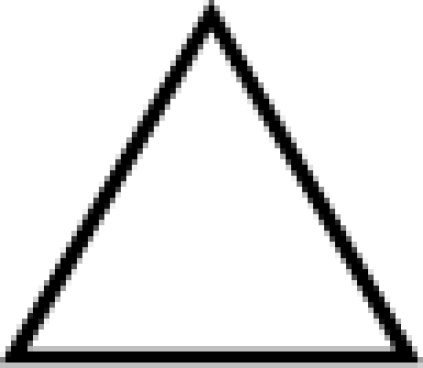
). The L value was measured once a day on the same skin area with a Chromameter CR-2300d. Averaged L values (n = 8) with an error bar of SD are plotted over the experimental period. A value of *p* < 0.01 by Student’s *t*-test by comparing the data with that of control at the same time was considered statistically significant.

In order to more closely approximate human usage, we used mice as a second *in vivo* animal model to investigate the depigmenting activity of RK. The skin of mice is more comparable to human skin than the skin of zebrafish is. After shaving, mice were treated with 0.2% RK or 2% RK in Vaseline and the skin lightening index was recorded. For this *in vivo* study, we used hydroquinone (HQ) as a positive standard, as it is the most widely used agent for skin depigmentation therapy around the world (Figure 6). Within one week of treatment, the degree of skin whitening was significantly increased in mice treated with 0.2% or 2% RK, compared to the control, and this increase continued until the end of the experiment. The depigmenting activity of 2% RK was comparable to that of 2% HQ. In our results, RK caused significant skin-whitening effect on mice skin at as low as a concentration of 0.2%. In contrast, Fukuda *et al*. ever investigated skin-whitening activity of RK on mice skin, and found RK had only weak skin-whitening activity even at as high as a concentration of 20% [17]. We suggest that the reasons for the different results of the two studies are due to the usage of different evaluating methods. Fukuda *et al.* used non-shaved mice to perform their experiment and evaluated the skin-whitening activity by visual observation only. Non-shaved mice have a higher barrier for drug penetration, and the skin-whitening activity is harder to be detected by naked eyes. In contrast, we used shaved mice as the experimental animal and a derma-spectrophotometer to evaluate the skin-whitening activity on mice skin, and hence could detect the skin-whitening activity of RK on mice skin in lower concentrations.

Safety is an important requirement for melanogenesis inhibitors used in cosmetic products. RK is one of the most common natural flavoring components in the food industry. In 1965, the FEMA placed RK on GRAS status. RK has also been widely used in perfumery and in cosmetics, to impart a fruity aroma. It is generally used in jasmine, tuberose, and gardenia fragrance formulas. It also can be used as a fixer in fruity flavorings. Thus, RK generates little concern about safety for human usage with the exception of workers engaged in RK manufacturing [18]. Based on our findings reported here, RK would appear to have high potential for use in the cosmetics industry.

## 3. Experimental Section

### 3.1. Materials

3-(4,5-Dimethylthiazol-2-yl)-2,5-diphenyltetrazolium bromide (MTT), arbutin, Triton X-100, phenylmethylsulfonyl fluoride (PMSF), l-DOPA, dimethyl sulfoxide (DMSO), trypsin/EDTA, synthetic melanin, 1-phenyl-2-thiourea (PTU), hydroquinone (HQ) and 3-isobutyl-1-methylxanthin (IBMX) were purchased from Sigma (St. Louis, MO). Vaseline was bought from a local store. Anti-tyrosinase antibodies (#62914) and protease inhibitor cocktail were obtained from Abcam (Cambridge, MA, USA). Anti-β-actin antibodies (#3662) were purchased from Bio Vision (Irvine, CA). All other chemicals were obtained from Tokyo Chemical Industry (Tokyo) and were of analytic reagent grade.

### 3.2. Isolation of RK from R. officinale

Dried powdered root and rhizomes of *R. officinal*e (5.5 kg) were extracted four times with 95% ethanol at room temperature. After removal of the solvent by evaporation, the residue (1.93 kg) was dissolved in methanol-water (9.5:0.5) and partitioned with *n*-hexane. The methanol (95%) was removed by evaporation and the residue was then suspended in water and partitioned with ethyl acetate (385.0 g). The ethyl acetate layer was subjected to silica gel column chromatography and eluted with *n*-hexane-ethyl acetate (9:1, 7.5:2.5, 1:1, 2.5:7.5), ethyl acetate, and ethyl acetate-methanol(9:1, 1:1), successively. Each fraction collected from the column was monitored by thin-layer chromatography and the similar fractions were combined to produce 4 fractions. The fr. 1 was further purified by a silica gel and eluted with *n*-hexane-ethyl acetate (9:1, 7.5:2.5, 1:1, 2.5:7.5) to isolate RK (49.2 mg). Their structures were confirmed by NMR and mass spectra analysis.

RK: colorless needles crystal; m.p. 82–84 °C; ESI/MS *m/z*: 163 [M-H]^+^; ^1^H-NMR (CDCl_3_, 500 MHz) δ: 2.14 (3H, s, H-1), 2.74 (2H, t, *J* = 7.5 Hz, H-3), 2.82 (2H, t, *J* = 7.5 Hz, H-4), 6.75 (2H, d, *J* = 8.5 Hz, H-3′,5′), 7.04 (2H, d, *J* = 8.5 Hz, H-2′,6′); ^13^C-NMR (CDCl_3_, 125 MHz) δ: 209.0 (C-2, C=O), 154.0 (C-4′), 132.8 (C-1′), 129.4 (C-2′, 6′), 115.3 (C-3′, 5′), 45.4 (C-3), 30.1 (C-4), 28.9 (C-1). These data were compared with literature values [12].

### 3.3. Cell Cultures and Drug Treatments

Mouse B16 melanoma cells (4A5) were obtained from the Bioresources Collection and Research Center (BCRC, Food Industry Research and Development Institute, Hsinchu). The cells were cultured in Dulbecco’s modified Eagle’s medium (DMEM) supplemented with 10% (v/v) fetal bovine serum at 37 °C in a humidified, CO_2_-controlled (5%) incubator. The cells were seeded at an appropriate cell density in a 24-well or a 6-well plate. After 1 d of incubation, the cells were treated with various concentrations of drugs in the absence or presence of a stimulation agent (100 μM of IBMX) for another 2 d. Thereafter, the cells were harvested and used for various assays.

### 3.4. Measurements of Cell Viability

MTT assays were performed to determine cell viability. After the cells were incubated with the drugs for 48 h, the culture medium was removed and replaced with 1 mg/mL MTT solution dissolved in phosphate-buffered saline (PBS) for a further 2 h incubation. The MTT solution was then removed, DMSO was added, and the absorbance of the dissolved formazan crystals was determined at 570 nm.

### 3.5. Fontana-Masson Stain

At the end of cell culture, the cells were harvested and washed twice with PBS. Fontana-Masson staining of B16 cells was conducted using a Fontana-Masson stain Kit (ScyTek Lab., Logan, UT, USA) according to the manufacturer’s instructions. The Fontana-Masson Stain Kit is intended for use in the visualization of melanin in cells, where cell nuclei, cytoplasm and melanin would display red, light pink, and black, respectively, after staining. The staining cells were photographed under a phase-contrast microscopy equipped with a digital camera.

### 3.6. Determination of Melanin Content

At the end of cell culture, the cells were harvested and washed twice with PBS. The pelleted cells were lysed in cold lysis buffer (20 mM sodium phosphate (pH 6.8), 1% Triton X-100, 1 mM PMSF, 1 mM EDTA). After centrifugation at 15,000 × g for 15 min, the melanin pellets were dissolved in 1 N NaOH containing 20% DMSO for 1 h at 95 °C. The absorbance at 490 nm was measured, and the melanin content was calculated against a known standard of synthetic melanin. The protein content in the supernatant was determined using the Bradford assay. The specific melanin content was adjusted by the amount of protein in the same reaction.

### 3.7. Measurements of Cellular Tyrosinase Activity

Tyrosinase activity in B16 cells was examined by measuring the rate of oxidation of l-DOPA. The drug-treated cells were washed with ice-cold PBS and lysed with 20 mM phosphate buffer (pH 6.8) containing 1% Triton X-100 and 1 mM PMSF. Detergent was used to release the membrane-bound tyrosinase from the melanosomes. Cells were then disrupted by freezing and thawing. The lysates were centrifuged at 15,000 × g for 15 min. The protein content in the supernatant was determined by the Bradford assay, with BSA as the protein standard. Tyrosinase activity was then determined as follows: 1 mL of the reaction mixture contained 50 mM of phosphate buffer (pH 6.8), 2.5 mM of l-DOPA, and 500 μg of the supernatant protein. After a 15 min reaction at 37 °C, dopachrome formation was monitored by measuring absorbance at 475 nm. One unit of tyrosinase activity was defined as the amount of enzyme protein that catalyzed the formation of 1 μmole of dopachrome in 1 min. The amount of dopachrome in the reaction was calculated by the Lambert-Beer Law using a molar extinction coefficient for dopachrome of 3600 M^−1^·cm^−1^ [19]. The specific tyrosinase activity was normalized with protein content in the reaction.

### 3.8. Measurements of Cell-free Tyrosinase Activity

The crude enzyme source of solubilized tyrosinase was prepared as described above, with the exception that untreated cells were used in this measurement. To determine the inhibitory effect of RK toward cell-free tyrosinase activity, the reaction mixture contained 50 mM phosphate buffer (pH 6.8), 2.5 mM of l-DOPA, the tested concentration of the drug, and 500 μg of the supernatant protein with a total volume of 1 mL of crude tyrosinase. After incubation at 37 °C for 15 min, dopachrome formation was monitored by measuring the absorbance at 475 nm. The relative tyrosinase activity was obtained by dividing the enzyme activity of the reaction mixture containing a drug by that without drugs.

### 3.9. Tyrosinase Zymography

l-DOPA staining assay was performed as our previous reported [20]. The cells were washed 3 times in ice-cold PBS, and lysed in cold lysis buffer (20 mM sodium phosphate (pH 6.8), 1% Triton X-100, 1 mM PMSF) containing a protease inhibitor cocktail (Abcam, Cambridge). An aliquot of the lysate was used to determine the protein content with a Bradford assay using BSA as the standard. The proteins (100 μg) were mixed with sampling buffer (no β-mercaptoethanol or heating) and separated using 10% SDS-polyacrylamide gel electrophoresis. Gel containing tyrosinase activity was rinsed in 200 mL of 100 mM sodium phosphate buffer (pH 6.8) and equilibrated at room temperature with gentle shaking. After 30 min, the rinse buffer was replaced with fresh buffer. After repeating the rinse procedure once again, the gel was transferred into 200 mL of a staining solution containing the rinse buffer supplemented with 5 mM l-DOPA, and incubated in the dark at 37 °C for 1 h. Tyrosinase activity was visualized in the gel as a dark melanin-containing band. The signal intensity of each band was quantified with a densitometer system GS-700 (Bio-Rad, Hercules, CA) equipped with an integrator.

### 3.10. Western Blot Analysis

The cells were washed 3 times in ice-cold PBS and lysed in cold lysis buffer (20 mM sodium phosphate (pH 6.8), 1% Triton X-100, 1 mM PMSF, 1 mM EDTA) containing protease inhibitors cocktail (Abcam). An aliquot of the lysate was used to determine the protein content with a Bradford assay using BSA as the standard. The proteins (100 μg) were separated on 10% SDS-polyacrylamide gel electrophoresis and blotted onto polyvinyl difluoride (PVDF) membranes (MP Biomedicals Co., Irvine, CA, USA). The membranes were blocked with 5% non-fat skim milk in TBS-T buffer. Tyrosinase and β-actin (as an internal control) were detected using a rabbit polyclonal antibodies and mouse monoclonal anti-β-actin antibodies, respectively. The membranes were further incubated with horseradish peroxidase-conjugated secondary antibody. All bound antibodies were then detected using an Amersham ECL system (Amersham Pharmacia Biotech, Piscataway, NJ, USA). The signal intensity of each band was quantified with a densitometer system GS-700 (Bio-Rad) equipped with an integrator, and normalized with that of the internal control. The relative tyrosinase protein content was calculated by dividing the normalized data by that from the IBMX-stimulated control reaction.

### 3.11. Quantitative Real-time Reverse Transcription Polymerase Chain Reaction (Real Time qRT-PCR)

Real time qRT-PCR were performed on the ABI 7500 Real Time PCR system (Applied Biosystems, Foster, CA) using Fast SYBR^®^ Green Master Mix (Applied Biosystems).Total RNA was extracted using an RNeasy^®^ mini Kit (Qiagen, Valencia, CA) according to the manufacturer’s instructions. The quality of the total RNA sample was evaluated by determining the OD_260_/OD_280_ ratio. To prepare a cDNA pool from each RNA sample, total RNA (2 μg) was reverse transcribed at 42 °C for 90 min in the presence of oligo(dT) primers (MD Bio., Taipei) and reverse transcriptase (Roche Molecular Biochemicals, Mannheim). The oligonucleotides primers for mouse tyrosinase (forward, 5′-GGCCAGCTTTCAGGCAGAGGT-3′; reverse, 5′-TGGTGCTTCATGGGCAAAATC-3′) and mouse glyceraldehyde-3-phosphate dehydrogenase (GAPDH) as an internal control (forward, 5′-ACCACAGTCCATGCCATCAC-3′; reverse, 5′-TCCACCACCCTGTTGCTGTA-3′) were used. After the initial incubation of 2 min at 50 °C, the cDNA was denatured at 95 °C for 10 min followed by 40 cycles of PCR (95 °C, 15 s, 60 °C, 60 s). All results were obtained from at least three independent experiments. The mRNA level of tyrosinase was normalized using GAPDH as an internal control. The relative tyrosinase mRNA content was calculated by dividing the normalized data by that from the IBMX-stimulated control reaction.

### 3.12. Determinations of Depigmenting Activity in Zebrafish

Evaluation of depigmenting activity in a zebrafish model system was assayed according to Choi *et al.* [16]. The study was approved by the Ethics Committee of National University of Tainan (Approval number: 9804). Adult zebrafish were obtained from a commercial dealer and kept in acrylic tanks with a 14/10 h light/dark cycle at 28 °C. Synchronized embryos were obtained from natural spawning induced in the morning by turning on the light. Test compounds were dissolved in 0.1% DMSO and then added to the embryo medium from 9 to 48 hpf (hours post fertilization). The developed zebrafish larvae were photographed with a digital camera under a stereomicroscope, then sonicated in cold lysis buffer (20 mM sodium phosphate (pH 6.8), 1% Triton X-100, 1 mM PMSF, 1 mM EDTA) containing protease inhibitors cocktail. An aliquot of the lysate was used to determine the protein content with a Bradford assay using BSA as the standard. The lysate was clarified by centrifuging at 10,000 × g for 10 min. The melanin precipitation in each tube was photographed with a digital camera and then resuspended with 0.2 mL of 1 N NaOH/20% DMSO at 95 °C for 1 h. The absorbance at 490 nm was measured, and the melanin content was quantified against an authentic standard of synthetic melanin. The specific melanin content was adjusted by the amount of protein in the same reaction.

For the tyrosinase activity assay, 250 μg of total protein in the lysate was added into a reaction mixture containing 50 mM phosphate buffer (pH 6.8) and 2.5 mM of l-DOPA. After incubation at 37 °C for 60 min, dopachrome formation was monitored by measuring the absorbance at 475 nm. The specific tyrosinase activity was normalized with the protein content in the reaction.

### 3.13. Determinations of Depigmenting Activity in Mice

Depigmenting activity was evaluated in a mouse model system using a modified protocol of Tai *et al.* [21]. The study was approved by the Ethics Committee of National Formosa University (Approval number: 9901). Five-week-old mice (C57BL/6J), weighing around 20 to 25 g, were obtained from the National Animal Laboratory Center, Taipei. Throughout the experiment, animals were housed in stainless steel cages in an air-conditioned room with temperature maintained at 25 to 28 °C and with alternating day and night cycles of 12 h. The animals were acclimatized for 7 days prior to the experiment. After shaving their hair, the animals were given 1 day’s rest. Gel samples containing RK or HQ were prepared by dispersing the drugs in Vaseline. A total of thirty-two mice were equally divided into four groups and each group was smeared twice daily with 0.1 g Vaseline (control), 2% HQ in Vaseline, 0.2% or 2% RK in Vaseline. The applications continued for 3 weeks and the skin-whitening index (L value) was measured on the same skin area every day with a Chromameter CR-2300d (Konica Minolta, Osaka), which is a colorimetric instrument that uses a Xenon lamp as a light source and is connected to a computer. The light reflected perpendicular to the skin was collected by photodetectors with colored filters for a tristimulus color analysis at 450, 560, and 600 nm, using the L*a*b* system, according to the CIE color systems [22]. We considered only the L parameter. The L-value gives the relative brightness, ranging from total black (L = 0) to total white (L = 100). After calibration against a white plate, the 8-mm probe was applied to the skin simply by the weight of the instrument. After the shutter button was pushed, the results were immediately read on the monitor. The initial skin-whitening index L-value was taken from the skin of each mouse before application of the tested substances. During the measurement, the photoreceivers were placed perpendicularly on the skin with minimal pressure.

### 3.14. Statistical Analysis

All of the data in the present study were obtained as averages of experiments that were performed at least in triplicate and are expressed as means ± SD Statistical analysis was performed by the Student’s *t* test. A value of *p* < 0.05 or *p* < 0.001 was considered to be statically significant.

## 4. Conclusions

This study demonstrated that RK from *R. officinale* inhibited melanogenesis through a post-transcriptional regulation of tyrosinase gene expression in cultured B16 melanoma cells. In addition, RK also inhibited melanogenesis of skin in both zebrafish and mice. Based on our findings reported here, RK would appear to have high potential for use in the cosmetics industry.
